# Relationship between Cerebral Microbleeds and Liver Stiffness Determined by Transient Elastography

**DOI:** 10.1371/journal.pone.0139227

**Published:** 2015-09-30

**Authors:** Young Dae Kim, Dongbeom Song, Ji Hoe Heo, Seung Up Kim, Beom Kyung Kim, Jun Yong Park, Do Young Kim, Sang Hoon Ahn, Kwang Joon Kim, Kwang-Hyub Han

**Affiliations:** 1 Department of Neurology, Yonsei University College of Medicine, Seoul, Korea; 2 Department of Internal Medicine, Institute of Gastroenterology, Yonsei University College of Medicine, Seoul, Korea; 3 Severance Executive Healthcare Clinic, Yonsei University Health System, Seoul, Korea; 4 Severance Check-up Severance Hospital, Yonsei University Health System, Seoul, Korea; Charité University Medicine Berlin, GERMANY

## Abstract

**Background & Aims:**

Liver fibrosis is a multifactorial disease that can affect the development of cerebral small vessel diseases (SVDs) including cerebral microbleeds (CMBs), leukoaraiosis, and silent infarctions. Transient elastography can accurately assess the degree of liver fibrosis by measuring liver stiffness (LS). In the present study, we investigated the association between SVDs and LS values.

**Methods:**

We recruited 300 participants (mean age 56 years, 170 men) who underwent a comprehensive medical health check-up between January 2011 and December 2012. Transient elastography was taken on the right lobe of the liver through intercostal space with patients lying in the dorsal decubitus position with the right arm in maximal abduction. Mild and significant fibrosis were defined as LS values >5.6 and >8.0 kPa, respectively. The presence of each SVD was determined using the FLAIR, GRE MR imaging as well as T1-, T2-weighted MR images. We tested whether the presence and burden of each type of SVD were different by LS values.

**Results:**

Of the different types of SVDs, only the presence (p = 0.001) and number of CMBs (p<0.001) were positively associated with LS values. Multivariate analysis revealed that significant fibrosis (>8.0 kPa) was an independent predictor of CMBs (odds ratio 6.079, 95% confidence interval 1.489–24.819, p = 0.012). However, leukoaraiosis and silent infarctions were not associated with LS values (all p>0.05).

**Conclusions:**

The degree of liver fibrosis, as assessed using transient elastography, was independently associated with the presence and burden of CMBs in healthy, asymptomatic participants. Understanding the link between the brain and liver may advance future research on the pathomechanisms of CMBs.

## Introduction

Cerebral small vessel disease (SVD) commonly occurs in the general population, especially in individuals over 60 years of age [1, 2]. Different types of SVD include leukoaraiosis, silent infarctions, and cerebral microbleeds (CMBs); leukoaraiosis refers to the rarefaction of white matter, silent infarction refers to ischemic damage in perforating territory without clinical stroke, and CMBs refer to previous extravasation of blood. All types of SVD are believed to be associated with clinical stroke, cognitive dysfunction, or vascular dementia [1, 3, 4]. In particular, the presence and burden of CMBs is strongly associated with the development of intracranial hemorrhage (ICH) [5, 6]. Furthermore, recent studies suggest a positive association between the burden of CMBs and other organ diseases such as pulmonary or renal diseases [7, 8]. Thus, it is of paramount importance to reveal potential links between cerebral SVD and the condition of other major organs.

Liver fibrosis is a multifactorial disease associated with systemic inflammation, insulin resistance, and arterial stiffness [9, 10], all of which can affect cerebral small vessels. Thus, a significant correlation may exist between the severity of cerebral SVD and liver fibrosis. To date, liver biopsy has been the gold standard for assessing the severity of liver fibrosis with acceptable safety [11]. However, liver biopsy can cause discomfort and involves rare but potentially life-threatening complications and sampling errors [12, 13]. Recently, the measurement of liver stiffness (LS) using transient elastography (TE; FibroScan^®^; EchoSens, Paris, France) has been introduced as a promising noninvasive approach for assessing the degree of liver fibrosis with considerable accuracy and reproducibility [14, 15].

The aim of this study was to determine whether the severity of cerebral SVD depends on the degree of liver fibrosis, as reflected by LS values using TE, in healthy, asymptomatic individuals undergoing a comprehensive medical health check-up.

## Patients and Methods

### Participants

We recruited 350 participants who underwent a comprehensive medical health check-up between January 2011 and December 2012 at Severance Check-up Severance Hospital, Yonsei University College of Medicine, Seoul, Korea. Exclusion criteria were as follows: (1) LS measurement failure (i.e., no valid shots); (2) invalid LS measurement; (3) no available brain magnetic resonance imaging (MRI) data; (4) chronic viral hepatitis; (5) heavy alcohol ingestion in excess of 40 g/day for more than 5 years, (6) right-sided heart failure; (7) pregnancy; and (8) other insufficient clinical, laboratory, and imaging data. Based on these exclusion criteria, 50 participants were excluded; data from the remaining 300 participants were included in the final statistical analysis. This study was approved by the Institutional Review Board of Severance Hospital, Yonsei University Health System. Informed consents were not required for this retrospective study.

During admission for check-up, complete medical examinations, laboratory tests, and imaging were performed as screening evaluations. Demographic and anthropometric data such as age, gender, history of hypertension, diabetes, hypercholesterolemia, alcohol consumption (g/week), body mass index, waist-hip ratio, and vital signs were collected. Laboratory tests included serological tests for hepatitis B virus surface antigen and hepatitis C virus antibody, a complete blood cell count, fasting glucose, aspartate aminotransferase, alanine aminotransferase, total bilirubin, alkaline phosphatase, international normalized ratio, activated prothrombin time, and lipid profiles (total cholesterol, triglyceride, high-density lipoprotein, and low-density lipoprotein cholesterol). Data for concomitant medications were also collected.

### LS Measurement Using TE

All participants underwent TE using an M probe. TE was performed on the right lobe of the liver through the intercostal space with the participant lying in the dorsal decubitus position with his or her right arm in maximal abduction. A single experienced technician blind to the participants’ clinical information performed all TE examinations. TE results were expressed as kilopascals (kPa) for LS. The interquartile range (IQR) was defined as the intrinsic variability of LS values corresponding to the interval of LS results containing 50% of the valid measurements between the 25th and 75th percentiles. The median value of the successful measurements was selected as representative of LS values for a given participant. As an indicator of variability, the ratio of the IQR to the median (IQR/M) of LS values was calculated. LS measurement failure was recorded when no value was obtained after at least 10 shots (i.e., valid shots = 0). A reliable LS value was defined by the following three criteria: (1) at least 10 valid shots; (2) a success rate (i.e., the ratio of valid shots to the total number of shots) of at least 60%; and (3) an IQR less than 30% of the median LS value (IQR/M<30%) [16, 17].

### Determination of LS Cutoff Values

The upper cutoff for normal LS values was considered 5.6 kPa based on a previous Korean study reporting a normal range of 3.3–5.6 kPa for healthy individuals without significant variation with age [18]. According to a previous study [19], we considered 8.0 kPa as the cutoff value representing the presence of significant liver fibrosis. Finally, we categorized LS values into three groups: <5.6 kPa for no fibrosis, 5.6–8.0 kPa for mild fibrosis, >8.0 kPa for significant fibrosis.

### Determination of SVD

The presence of SVD such as CMBs, leukoaraiosis, silent infarction was determined using the baseline MRI. All MRI examinations were performed using a 3.0T MRI system (Achieva 3.0T, Philips Medical Systems, Best, Netherlands or MAGNETOM 3.0T, Trio A Tim System, Siemens, Germany). Brain MRI images were obtained parallel to the orbitomeatal line using the following parameters: TR/TE 9000/120 ms, pixel spacing 0.449 mm/0.449 mm, FOV 230×230 mm, and slice thickness 5 mm for fluid-attenuated inversion recovery (FLAIR); TR/TE 9000/100 ms, pixel spacing 0.240 mm/0.240 mm, FOV 230×230 mm, and slice thickness 5 mm for T2 weighted images (T2); and TR/TE 600/16 ms, pixel spacing 0.449 mm/0.449 mm, FOV 250×250 mm, and slice thickness 5 mm for gradient recalled echo (GRE) imaging.

CMBs were defined as punctuate hypointense lesions <10 mm in size and located in lobar (cortex, subcortex, and white matter), deep (basal ganglia and thalamus), or infratentorial (brain stem and cerebellum) regions on GRE MRI images, based on previously reported methods [1]. Hypointense lesions on GRE MRI images located in cerebral vessels, calcifications, air-bone interfaces, and partial volume artifacts at the edges of the cerebellum were excluded. Small hypointense lesions on GRE MRI images <3 mm in size were also excluded because these lesions could be dilated perivascular spaces, demyelination areas, or gliosis.

We also determined the severity of leukoaraiosis and silent infarction in our study sample. The extent of leukoaraiosis was investigated on FLAIR images of periventricular white matter or deep white matter according to the Fazekas scoring system [20]. Leukoaraiosis in periventricular white matter was categorized as: grade 0, absent; grade 1, caps or pencil-thin lining; grade 2, smooth halo; or grade 3, irregular periventricular hemorrhage extending into deep white matter. Leukoaraiosis in deep white matter was categorized as: grade 0, absent; grade 1, punctuate foci; grade 2, beginning confluence of foci; or grade 3, large confluent areas. Silent infarctions were defined as high signal intensity on FLAIR or T2-weighted images and iso- or low signal intensity on T1-weighted images in participants without previous clinical stroke, based on a previous report [21]. Lesions with <3 mm diameter were not considered as silent infarctions because of the risk of misdiagnosis with dilatation of the perivascular space, demyelination, or gliosis.

The presence of CMBs, leukoaraiosis, and silent infarctions on MRI images was investigated by two neurologists (Y.D.K. and D.S.) blind to participant clinical information. Final classifications were made by consensus between the two neurologists.

### Statistical Analysis

Statistical analyses were performed using the Windows SPSS software package (version 21.0, Chicago, IL, USA). To determine statistically significant differences in continuous variables between participants with and without SVD, independent samples t-tests or Mann-Whitney tests were used, whereas differences in categorical variables were evaluated using chi-square tests or Fisher’s exact tests, as appropriate. Increases in the degree of SVD (either CMBs, leukoaraiosis, or silent infarctions) depending on LS values were assessed using linear-by-linear tests. Differences in mean LS values depending on the number of CMBs were investigated using analysis of variance (ANOVA) with Scheffe post-hoc tests. To identify independent predictors of the presence of different types of SVD, multivariate logistic regression analysis was performed including variables with p<0.1 in univariate analysis (model 1). We also performed an exploratory multivariate analysis entering all cardiovascular risk factors and all variables with p<0.1 in univariate analysis (model 2) as well as age and sex. A p value of <0.05 was considered statistically significant.

## Results

### Baseline Characteristics

Baseline characteristics of the study sample are shown in **Table 1**. The mean age of participants was 56.0 ± 11.2 years, and 170 (56.7%) were male. Among vascular risk factors, smoking was the most common (37.0%), followed by hypertension (30.7%) and diabetes (12.7%). The antithrombotics were used in 58 (19.3%) patients (antiplatelet agent on 56 (18.7%) patients, oral anticoagulation on 2 (0.7%) patients). Mean aspartate and alanine aminotransferase values were 0.4±0.2 μkat/L and 0.4±0.2 μkat/L, respectively. The mean LS value was 4.8±2.3 kPa. According to the predetermined definition of fibrotic burden, 45 (15.0%) participants had mild fibrosis, and 12 (4.0%) participants had significant fibrosis.

**Table 1:** Baseline characteristics.

Variables	Total population (n = 300)	CMB (-) (n = 236)	CMB (+) (n = 64)	p value
Age	56.0 ± 11.2	54.9 ± 11.0	60.3 ± 11.2	0.001
Male sex	170 (56.7)	126 (53.4)	44 (68.8)	0.028
Hypertension	92 (30.7)	71 (30.1)	21 (32.8)	0.675
Diabetes	38 (12.7)	28 (11.9)	10 (15.6)	0.422
Hyperlipidemia	30 (10.0)	24 (10.2)	6 (9.4)	0.851
Smoking	111 (37.0)	88 (37.3)	23 (35.9)	0.843
Atrial fibrillation	5 (1.7)	3 (1.3)	2 (3.1)	0.304
Previous ischemic heart disease	22 (7.3)	14 (5.9)	8 (12.5)	0.074
Medication				
Antihypertensive medication	76 (25.3)	60 (25.4)	16 (25.0)	0.945
Antidiabetic medication	35 (11.7)	24 (10.2)	11 (17.2)	0.121
Statin	65 (21.7)	42 (17.8)	23 (35.9)	0.002
Antithrombotics	58 (19.3)	38 (16.1)	20 (31.2)	0.006
Alcohol consumption (g/week)	131.1 ± 296.5	121.3 ± 260.5	168.1 ± 405.2	0.271
Initial systolic blood pressure, mmHg	122.7 ± 15.8	121.8 ± 15.1	126.0 ± 18.0	0.062
Initial diastolic blood pressure, mmHg	79.8 ± 13.4	79.5 ± 13.8	80.8 ± 12.3	0.493
Body mass index	24 ± 3.2	24.1 ± 3.2	23.8 ± 3.0	0.503
Waist-hip ratio	0.9 ± 0	0.9 ± 0	0.9 ± 0	0.373
White blood cell, x109/L	5.6 ± 1.6	5.6 ± 1.6	5.6 ± 1.7	0.886
Hemoglobin, g/L	141.7 ± 15.6	141.7 ± 15.8	142.0 ± 14.7	0.864
Fasting glucose, mmol/L	5.7 ± 3.2	5.5 ± 1.2	6.6 ± 6.6	0.172
Blood urea nitrogen, mmol/L	5.6 ± 2.7	5.4 ± 2.8	6.0 ± 2.3	0.101
Serum creatinine, μmol/L	74.6 ± 49.2	69.3 ± 15.3	94.2 ± 100.6	0.053
Alkaline phosphatase, μkat/L	0.9 ± 0.2	0.9 ± 0.2	0.9 ± 0.2	0.943
Aspartate aminotransferase, μkat/L	0.4 ± 0.2	0.4 ± 0.1	0.4 ± 0.2	0.204
Alanine aminotransferase, μkat/L	0.4 ± 0.2	0.4 ± 0.2	0.4 ± 0.3	0.182
Total bilirubin, μmol/L	14.6 ± 19	15.0 ± 21.2	13.1 ± 5.9	0.489
Serum albumin, g/L	42.6 ± 2.5	42.7 ± 2.5	42.0 ± 2.7	0.072
Total cholesterol, mmol/L	4.9 ± 1.0	4.9 ± 1.0	4.8 ± 1.1	0.394
Triglyceride, mmol/L	1.3 ± 0.7	1.3 ± 0.7	1.4 ± 0.6	0.676
High density lipoprotein, mmol/L	1.2 ± 0.3	1.2 ± 0.3	1.2 ± 0.3	0.426
Low density lipoprotein, mmol/L	3.0 ± 0.9	3.0 ± 0.8	2.9 ± 1.0	0.369
Glycosylated hemoglobin, %	6.0 ± 0.9	6.0 ± 0.8	6.2 ± 1.2	0.092
International normalized ratio	0.9 ± 0.1	0.9 ± 0.1	0.9 ± 0.1	0.237
Activated promthrombin time, s	31.7 ± 4.1	31.8 ± 4.0	31.4 ± 4.3	0.454
Liver stiffness values (kPa)	4.8 ± 2.3	4.5 ± 1.5	5.8 ± 4.0	0.009
Degree of liver fibrosis				0.001
No fibrosis (<5.6 kPa)	243 (81.0)	198 (83.9)	45 (70.3)	
Mild fibrosis (5.6–8.0 kPa)	45 (15.0)	34 (14.4)	11 (17.2)	
Significant fibrosis (>8.0 kPa)	12 (4.0)	4 (1.7)	8 (12.5)	

Variables are expressed as mean ± SD or n (%).

CMBs indicates cerebral microbleeds; kPa, kilopascal.

### SVD Status

Of the study participants, 64 (21.3%) had CMBs in any location. Of these, most participants had a single CMB (n = 55, 18.3%), whereas six (2.0%) and three (1.0%) participants had two and three CMBs, respectively. CMBs in non-lobar areas were observed in 41 (13.7%) participants [subcortex: n = 29 (9.7%); infratentorial area: n = 14 (4.7%)], and lobar CMBs were observed in 23 (7.7%) participants. Leukoaraiosis was detected in 200 (66.7%) participants. Of those with periventricular white matter lesions, 122 (40.7%) were grade 1, 29 (9.7%) were grade 2, and 11 (3.7%) were grade 3. Of those with deep white matter lesions, 162 (54.0%) were grade 1, 10 (3.3%) were grade 2, and 7 (2.3%) were grade 3. Silent infarctions were observed in 26 (8.7%) participants.

### Association between Degree of Liver Fibrosis and Types of SVD

The presence of leukoaraiosis and silent infarctions were not significantly associated with LS values or the degree of liver fibrosis (all p>0.05, **Tables A and B in S1 File**). By contrast, participants with higher numbers of CMBs had significantly higher LS values (p<0.001); participants with 0, 1, 2, and 3 CMBs showed mean LS values of 4.5 ± 1.5, 5.0 ± 2.5, 6.5 ± 3.7, and 12.4 ± 10.7, respectively.

### Comparison between Participants with and without CMBs

Differences in baseline characteristics between participants with and without CMBs are shown in **Table 1**. Participants with CMBs were more likely to be older and of male gender, use statins or antithrombotics, and have higher LS values and degree of liver fibrosis (all p<0.05). Participants with previous ischemic heart disease, higher systolic blood pressure, and elevated levels of creatinine, serum albumin, or glycosylated hemoglobin tended to be more likely to have CMBs (p<0.1). Differences in other variables were not statistically significant (all p>0.1).

**Fig 1:**
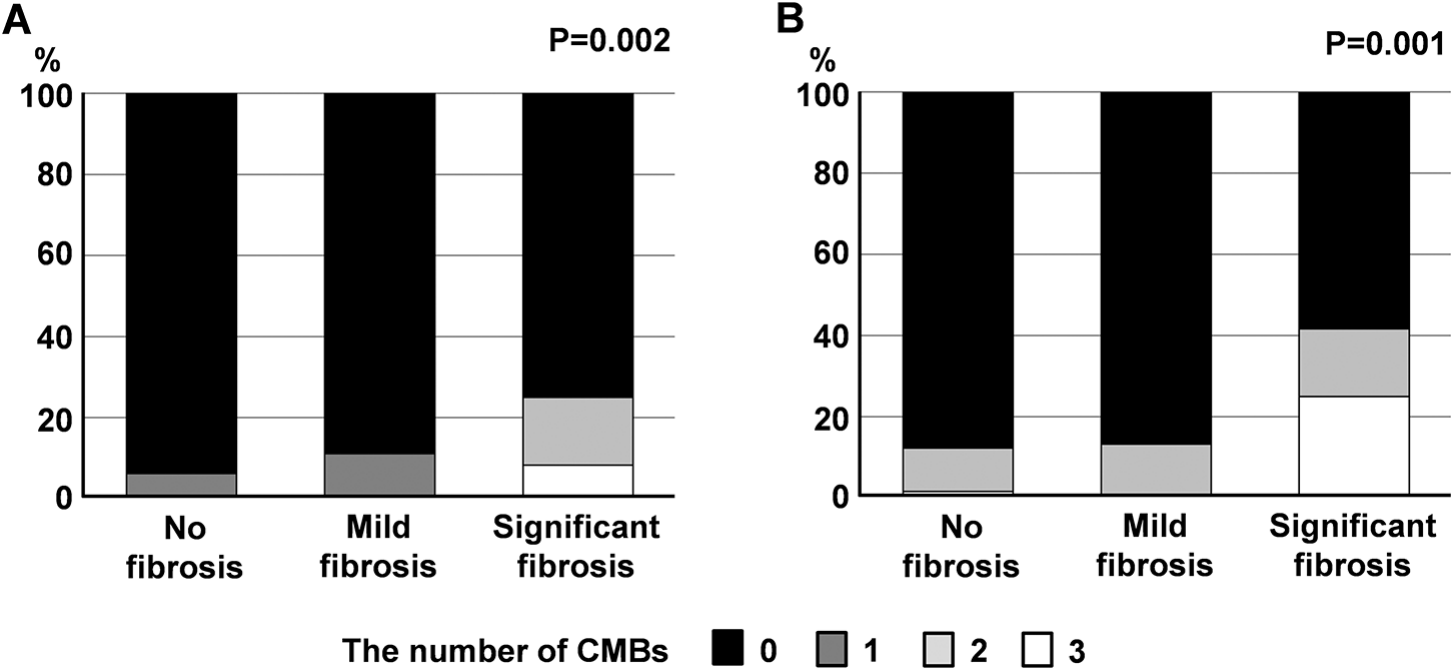
Increasing number of CMBs in lobar (A) and non-lobar (B) areas with higher LS values.

When we divided the study sample into three groups using pre-defined cutoff LS values, we found a significant positive association between the degree of liver fibrosis and the presence of CMBs (p = 0.001) regardless of their location (lobar, p = 0.015; non-lobar, p = 0.030). In terms of the burden of CMBs, we found a positive association between the degree of liver fibrosis and the number of CMBs (p<0.001) regardless of their location (lobar, p = 0.002; non-lobar, p = 0.001) (**Fig 1A and 1B**).

### Independent Predictors of the Presence of CMBs

Multivariate analysis demonstrated that significant fibrosis was an independent positive predictor of the presence of CMBs (odd ratio [OR] 6.165, 95% confidence interval [CI] 1.530–24.839, p = 0.011) after adjusting for variables with p<0.1 in univariate analysis (model 1), along with age (OR 1.033, 95% CI 1.000–1.066, p = 0.048) (**Table 2**). When cardiovascular risk factors were further adjusted, significant fibrosis remained an independent predictor of CMBs (OR 5.701, 95% CI 1.370–23.721, p = 0. 017) (model 2), along with male gender (OR 2.611, 95% CI 1.120–6.088, p = 0.026), and statin use (OR 2.195, 95% CI 1.060–4.542, p = 0.034).

**Table 2:** Independent predictors of the presence of CMBs.

Variables	Model 1*		Model 2^†^	
	Odd ratio (95% CI)	p value	Odd ratio (95% CI)	p value
Age	1.033 (1.000–1.066)	0.048	1.033 (0.999–1.067)	0.055
Male sex	1.886 (0.916–3.881)	0.085	2.611 (1.120–6.088)	0.026
Hypertension	-		0.505 (0.233–1.094)	0.083
Diabetes	-		0.454 (0.122–1.694)	0.240
Hypercholesterolemia	-		1.468 (0.516–4.172)	0.471
Smoking	-		0.564 (0.261–1.218)	0.145
Atrial fibrillation	-		1.568 (0.213–11.549)	0.659
Previous ischemic heart disease	0.942 (0.308–2.876)	0.916	0.922 (0.296–2.874)	0.889
Statin use	1.814 (0.912–3.609)	0.090	2.195 (1.060–4.542)	0.034
Antithrombotics use	1.165 (0.524–2.592)	0.708	1.613 (0.68–3.824)	0.278
Systolic blood pressure, per 1 mmHg	1.000 (0.980–1.021)	0.984	1.000 (0.975–1.025)	0.977
Diastolic blood pressure, per 1 mmHg	-		1.007 (0.981–1.035)	0.601
Serum creatinine, per 1 μmol/L	1.007 (0.995–1.018)	0.245	1.008 (0.997–1.019)	0.141
Serum albumin, per 1g/L	0.944 (0.832–1.071)	0.369	0.940 (0.825–1.070)	0.348
Glycosylated hemoglobin, per 1%	0.917 (0.646–1.301)	0.627	1.125 (0.706–1.792)	0.620
Degree of liver fibrosis				
No fibrosis (<5.6 kPa)	1		1	
Mild fibrosis (5.6–8.0 kPa)	1.214 (0.534–2.760)	0.643	1.326 (0.565–3.112)	0.516
Significant fibrosis (>8.0 kPa)	6.165 (1.530–24.839)	0.011	5.701 (1.370–23.721)	0.017

* Adjusted for age, sex, and the variables with p<0.1 in univariate analysis.

^†^ Adjusted for age, sex, the variables with p<0.1 in univariate analysis, and cardiovascular risk factors.

CMBs indicates cerebral microbleeds; CI, confidence interval; kPa, kilopascal.

### Sub-Group Analysis According to Age

Because CMBs are commonly found in individuals over 60 years of age [2], we further investigated potential differences in the relationship between CMBs and the degree of liver fibrosis in participants aged ≥60 years (n = 104, 45.7%) or <60 years (n = 196, 65.3%). The degree of liver fibrosis was positively associated with the presence of CMBs in participants aged ≥60 years (p = 0.008) and in those aged <60 years (p = 0.075). Multivariate analysis adjusting for all cardiovascular risk factors and significant variables with p<0.1 in univariate analysis also show a positive association between significant fibrosis and the presence of CMBs both in participants <60 years of age (p = 0.011) and in those ≥60 years of age (p = 0.055) (**Table 3**).

**Table 3:** Multivariate analysis of the presence of CMBs according to participant age.

	Age <60* (n = 196, 65.3%)		Age ≥60^†^ (n = 104, 45.7%)	
	Odd ratio (95% CI)	p value	Odd ratio (95% CI)	p value
Degree of liver fibrosis				
No fibrosis (<5.6 kPa)	1		1	
Mild fibrosis (5.6–8.0 kPa)	0.708 (0.184–2.729)	0.616	1.690 (0.445–6.413)	0.441
Significant fibrosis (>8.0 kPa)	10.686 (1.716–66.541)	0.011	8.696 (0.953–79.337)	0.055

* Adjusted for age, sex, all cardiovascular risk factors and significant variables with p<0.1 in univariate analysis (statin use).

^†^ Adjusted for age, sex, all cardiovascular risk and significant variables with p<0.1 in univariate analysis (statin use and blood urea nitrogen).

CMBs indicates cerebral microbleeds; CI, confidence interval; kPa, kilopascal.

## Discussion

We found that the prevalence of mild and significant liver fibrosis was 15.0% and 4.0%, respectively, and that greater liver fibrosis, as reflected by higher LS values, was independently associated with the presence and burden of CMBs in healthy, asymptomatic individuals undergoing a comprehensive medical health check-up. Leukoaraiosis or silent infarctions, which are ischemic types of cerebral SVD, were not associated with the degree of liver fibrosis. To the best of our knowledge, this is the first report showing a positive association between the degree of liver fibrosis and the burden of CMBs.

Although the exact mechanism is unclear, one possible explanation for the relationship between liver fibrosis and CMBs is that liver and brain may share common risk factors. Indeed, because cardiovascular risk factors and metabolic syndromes are the major driving forces for liver fibrosis and cerebral SVD [2, 22], liver fibrosis and cerebral SVD can exist simultaneously. However, we found that the association between CMBs and fibrotic burden was maintained even after adjusting for cardiovascular risk factors. Furthermore, previous studies show that chronic liver diseases such as non-alcoholic fatty liver diseases with a potential to progress to liver cirrhosis are independently associated with arterial stiffness, inflammation, and endothelial dysfunction in other organs which are also related to the development of CMBs [22–26]. These findings indicate that liver fibrosis may be a significant risk factor for the development of CMBs and that a direct link may exist between the liver and the brain.

In our study, leukoaraiosis and silent infarctions were not associated with the degree of liver fibrosis. However, previous studies suggest a close relationship between liver dysfunction and leukoaraiosis. For example, liver transplantation can reduce white matter lesion volume and lessen cognitive impairment [27, 28]. However, white matter lesions associated with liver cirrhosis are more widespread in later stages of the illness and might be caused by edematous changes rather than arteriosclerosis [29]. Thus, our results suggest that the subclinical stage of liver fibrosis has no impact on ischemic types of cerebral SVD. However, because the sample size of our study was relatively small, future large-scale studies are required to resolve this issue.

Interestingly, in terms of the location of CMBs, the degree of liver fibrosis was associated with the burden of not only non-lobar CMBs but also lobar CMBs, which may be a marker of cerebral amyloid angiopathy. Liver cirrhosis may be a risk factor for ICH due to coagulation abnormalities, thrombocytopenia, and low serum cholesterol, but these abnormalities are not sufficient for fully explaining the development of ICH in liver dysfunction [30]. Although there are no reports showing the association between the location of CMBs and liver dysfunction, previous reports show that the incidence of lobar ICH is more common than subcortical region in patients with liver cirrhosis [31, 32]. However, further studies are required to determine the association between the degree of liver fibrosis and lobar CMBs.

Together with the degree of liver fibrosis, statin use remained an independent predictor of CMBs within our study sample. Previous studies show that statin use may increase the risk of ICH [33]. Considering that CMBs are a prerequisite for ICH, the relationship between statin use and ICH suggests that statin use may be associated with CMBs before clinical ICH occurs.

Several issues in the present study remain unresolved. Although we consecutively enrolled participants, our study was a retrospective analysis and therefore may involve selection bias. Also, because most participants included in our study were healthy, asymptomatic individuals, thus resulting in a low rate of detection of high grade types of SVD such as leukoaraiosis, our results should be interpreted cautiously. However, using this study sample, we were able to investigate early connections between brain and liver pathology during the preclinical stage. Furthermore, although TE is an accurate tool for assessing the degree of liver fibrosis, histological information was not available in this study. Because all participants in our study were asymptomatic, it was not feasible to perform liver biopsy.

In conclusion, we demonstrated that harder livers are associated with a higher burden of CMBs. Understanding the link between the brain and the liver may advance research on the pathomechanism of CMBs and help establish optimal screening and treatment strategies.

## Supporting Information

S1 File Table A. Independent predictors of the presence of leukoaraiosis. Table B. Independent predictors of the presence of silent infarction. (DOCX)
