# Supplementary material for: Relationship between Cerebral Microbleeds and Liver Stiffness Determined by Transient Elastography
Source: PLoS One. 2015 Sep 30;10(9):e0139227. doi: 10.1371/journal.pone.0139227 (PMC4589390; doi:10.1371/journal.pone.0139227)
Supplement: S1 File — (DOCX) [file pone.0139227.s001.docx]

**Supplementary table 1. Independent predictors of the presence of leukoaraiosis**

| **Variables** | **Model 1^*^** |  |  | **Model 2^†^** |  |
| --- | --- | --- | --- | --- | --- |
|  | **Odd ratio (95% CI)** | **p value** |  | **Odd ratio (95% CI)** | **p value** |
| Age | 1.088 (1.051 - 1.125) | <0.001 |  | 1.094 (1.055 - 1.134) | <0.001 |
| Male sex | 0.586 (0.310 - 1.109) | 0.101 |  | 0.618 (0.263 - 1.450) | 0.269 |
| Hypertension | 1.446 (0.700 - 2.990) | 0.319 |  | 1.528 (0.723 - 3.232) | 0.267 |
| Diabetes | 5.641 (0.901 - 35.317) | 0.064 |  | 5.388 (0.858 - 33.834) | 0.072 |
| Hypercholesterolemia | - |  |  | 1.039 (0.368 - 2.939) | 0.942 |
| Smoking | - |  |  | 1.122 (0.488 - 2.581) | 0.786 |
| Atrial fibrillation | - |  |  | 0.199 (0.017 - 2.377) | 0.202 |
| Previous ischemic heart disease | 1.250 (0.289 - 5.411) | 0.765 |  | 1.257 (0.286 - 5.521) | 0.762 |
| Statin use | 2.162 (0.897 - 5.214) | 0.086 |  | 2.156 (0.887 - 5.243) | 0.090 |
| Antithrombotics use | 0.865 (0.327 - 2.286) | 0.770 |  | 0.865 (0.319 - 2.340) | 0.775 |
| Systolic blood pressure, per 1 mmHg | - |  |  | 0.988 (0.963 - 1.013) | 0.353 |
| Diastolic blood pressure, per 1 mmHg | 1.022 (0.998 - 1.046) | 0.078 |  | 1.030 (1.000 - 1.062) | 0.054 |
| White blood cell count, per 1x10^9^/L | 1.165 (0.956 - 1.421) | 0.130 |  | 1.129 (0.920 - 1.386) | 0.247 |
| Blood urea nitrogen, per 1 mmol/L | 1.029 (0.885 - 1.198) | 0.708 |  | 1.027 (0.888 - 1.187) | 0.724 |
| Aspartate aminotransferase, per 1µkat/L | 10.411 (0.807 - 134.354) | 0.073 |  | 8.548 (0.627 - 116.492) | 0.107 |
| Serum albumin, per 1 g/L | 0.920 (0.811 - 1.044) | 0.196 |  | 0.926 (0.815 - 1.052) | 0.236 |
| Low density lipoprotein, per 1 mmol/L | 0.934 (0.65 - 1.341) | 0.711 |  | 0.953 (0.645 - 1.409) | 0.810 |
| Glycosylated hemoglobin, per 1 % | 0.682 (0.406 - 1.146) | 0.149 |  | 0.697 (0.416 - 1.169) | 0.171 |
| activated promthrombin time, s | 0.956 (0.894 - 1.023) | 0.195 |  | 0.956 (0.893 - 1.023) | 0.191 |
| Degree of liver fibrosis |  |  |  |  |  |
| No fibrosis (<5.6 kPa) | 1 |  |  | 1 |  |
| Mild fibrosis (5.6-8.0 kPa) | 1.170 (0.46 - 2.975) | 0.742 |  | 1.187 (0.462 - 3.046) | 0.722 |
| Significant fibrosis (>8.0 kPa) | 0.974 (0.153 - 6.205) | 0.978 |  | 0.958 (0.150 - 6.125) | 0.964 |

^*^ Adjusted for age, sex, and the variables with p<0.1 in univariate analysis.

^†^ Adjusted for age, sex, the variables with p<0.1 in univariate analysis, and cardiovascular risk factors.

CI indicates confidence interval; kPa, kilopascal.

**Supplementary table 2. Independent predictors of the presence of silent infarction**

| **Variables** | **Model 1^*^** |  |  | **Model 2^†^** |  |
| --- | --- | --- | --- | --- | --- |
|  | **Odd ratio (95% CI)** | **P value** |  | **Odd ratio (95% CI)** | **P value** |
| Age | 1.057 (0.996 - 1.121) | 0.069 |  | 1.045 (0.980 - 1.114) | 0.180 |
| Male sex | 1.084 (0.321 - 3.662) | 0.897 |  | 1.281 (0.318 - 5.164) | 0.728 |
| Hypertension | 5.927 (1.715 - 20.48) | 0.005 |  | 7.515 (1.987 - 28.419) | 0.003 |
| Diabetes | 10.072 (2.056 - 49.351) | 0.004 |  | 11.298 (2.065 - 61.824) | 0.005 |
| Hypercholesterolemia | - |  |  | 1.199 (0.163 - 8.845) | 0.859 |
| Smoking | - |  |  | 0.604 (0.148 - 2.472) | 0.483 |
| Atrial fibrillation | - |  |  | 4.114 (0.244 - 69.374) | 0.326 |
| Previous ischemic heart disease | 1.780 (0.355 - 8.920) | 0.483 |  | 1.690 (0.304 - 9.380) | 0.549 |
| Statin use | 1.000 (0.286 - 3.496) | 1.000 |  | 0.852 (0.234 - 3.109) | 0.809 |
| Antithrombotics use | 0.756 (0.205 - 2.787) | 0.674 |  | 0.599 (0.143 - 2.506) | 0.483 |
| Systolic blood pressure, per 1 mmHg | 0.988 (0.953 - 1.024) | 0.496 |  | 0.997 (0.949 - 1.048) | 0.915 |
| Diastolic blood pressure, per 1 mmHg | - |  |  | 0.966 (0.899 - 1.038) | 0.348 |
| Waist-Hip ratio | 2.256 (0.000 - 834494.338) | 0.901 |  | 15.691 (0.000 - 10229800.433) | 0.687 |
| Fasting glucose, per 1 mmol/L | 1.442 (0.975 - 2.131) | 0.067 |  | 1.489 (0.984 - 2.253) | 0.060 |
| Serum albumin, per 1g/L | 0.798 (0.636 - 1.001) | 0.051 |  | 0.794 (0.631 - 0.999) | 0.049 |
| Total cholesterol, per 1 mmol/L | 0.580 (0.179 - 1.886) | 0.365 |  | 0.596 (0.173 - 2.045) | 0.410 |
| High density lipoprotein, per 1 mmol/L | 1.046 (0.100 - 10.896) | 0.970 |  | 0.83 (0.078 - 8.802) | 0.877 |
| Low density lipoprotein, per 1 mmol/L | 1.971 (0.474 - 8.200) | 0.351 |  | 1.919 (0.448 - 8.216) | 0.380 |
| Glycosylated hemoglobin, per 1 % | 0.782 (0.416 - 1.471) | 0.447 |  | 0.76 (0.394 - 1.466) | 0.413 |
| Degree of liver fibrosis |  |  |  |  |  |
| No fibrosis (<5.6 kPa) | 1 |  |  | 1 |  |
| Mild fibrosis (5.6-8.0 kPa) | 0.452 (0.104 - 1.958) | 0.288 |  | 0.420 (0.092 - 1.915) | 0.263 |
| Significant fibrosis (>8.0 kPa) | 0.727 (0.058 - 9.054) | 0.804 |  | 0.790 (0.054 - 11.655) | 0.864 |

^*^ Adjusted for age, sex, and the variables with p<0.1 in univariate analysis.

^†^ Adjusted for age, sex, the variables with p<0.1 in univariate analysis, and cardiovascular risk factors.

CI indicates confidence interval; kPa, kilopascal.
